# Associations between Physical Activity Level and Mental Health in the Spanish Population: A Cross-Sectional Study

**DOI:** 10.3390/healthcare10081442

**Published:** 2022-08-01

**Authors:** Ángel Denche-Zamorano, Sabina Barrios-Fernandez, Rafael Gómez-Galán, Juan Manuel Franco-García, Jorge Carlos-Vivas, María Mendoza-Muñoz, Jorge Rojo-Ramos, Alejandro Vega-Muñoz, Nicolás Contreras-Barraza, Konstantinos Gianikellis, Laura Muñoz-Bermejo

**Affiliations:** 1 Promoting a Healthy Society Research Group (PHeSO), Faculty of Sport Sciences, University of Extremadura, 10003 Cáceres, Spain; andeza04@alumnos.unex.es (Á.D.-Z.); jorgecv@unex.es (J.C.-V.); 2 Social Impact and Innovation in Health (InHEALTH), Faculty of Sports Sciences, University of Extremadura, 10003 Cáceres, Spain; rgomez@unex.es (R.G.-G.); jorgerr@unex.es (J.R.-R.); lauramunoz@unex.es (L.M.-B.); 3 Health, Economy, Motricity and Education (HEME) Research Group, Faculty of Sport Sciences, University of Extremadura, 10003 Cáceres, Spain; jmfrancog@unex.es; 4 Research Group on Physical and Health Literacy and Health-Related Quality of Life (PHYQOL), Faculty of Sport Sciences, University of Extremadura, 10003 Caceres, Spain; 5 Departamento de Desporto e Saúde, Escola de Saúde e Desenvolvimento Humano, Universidade de Évora, 7004-516 Évora, Portugal; 6 Public Policy Observatory, Universidad Autónoma de Chile, Santiago 7500912, Chile; alejandro.vega@uautonoma.cl; 7 Facultad de Economía y Negocios, Universidad Andres Bello, Viña del Mar 2531015, Chile; nicolas.contreras@unab.cl; 8 Biomecánica del Movimiento Humano y Ergonomía Research Group (BIOERGON), Faculty of Sport Sciences, University of Extremadura, 10003 Cáceres, Spain; kgiannik@unex.es

**Keywords:** exercise, health, psychology, mental disorders, health survey

## Abstract

Physical inactivity and sedentary lifestyles appear to be critical factors in developing mental health problems, including depression, anxiety, and other diseases in developed societies. This study analysed the associations between physical activity level (PAL) and mental health using the Goldberg General Health Questionnaire (GHQ12) in the Spanish population before the COVID-19 pandemic. A cross-sectional design, based on data from the Spanish National Health Survey (ENSE 2017), the last health survey before the pandemic, was carried out with 17,641 participants. Data did not follow a normal distribution, so non-parametric tests were used to analyse intergroup differences, differences at baseline and post hoc, and correlations between variables. Associations were found between the PAL, mental health and all its dimensions. The groups that performed moderate and intense PAL showed lower values in the GHQ12 questionnaire than those who walked or were inactive. Thus, higher PAL was associated with better mental health indicators, including successful coping, self-esteem and stress. This study provides a framework to compare outcomes between the pre- and post-pandemic periods, as the ENSE is performed every five years.

## 1. Introduction

Mental disorders are the second leading cause of illness globally [1]. Anxiety and depression are the most prevalent mental disorders in the general population [2]. Mental health problems are associated with a higher prevalence of chronic diseases [3], poor adherence to medical treatment [4], increased morbidity [5,6,7] and premature mortality [8]. As an example, the 12-month prevalence of anxiety disorders was 13.4% in Europe (69.1 million people), costing more than 74 billion euros [9], and 22% in the United States [10]. Moreover, the results of international and national studies have demonstrated their great economic and social impact [11,12]. In Spain, mental health disorders are considered, together with neurological diseases, the leading cause of disability among non-infectious diseases [13]. The psychological distress responsible for a great number of mental disorders is defined as “a series of symptoms and experiences of a person’s inner life that are commonly considered to be troubling, confusing or out of the ordinary” [14]. Moreover, the psychiatric morbidity in Spain was 22.2% in 2006 and 22.1% in 2011, with differences according to geographical areas, sex, and economic situation, among others [15].

Psychological distress symptomatology implies a breakdown in daily functioning, including anxiety, depression, loss of self-confidence and the inability to make decisions, among others. In different national health surveys, mental health status is usually identified with self-administered tools, such as the General Health Questionnaire (GHQ-12) [16]. The GHQ-12 defines psychiatric morbidity by classifying subjects as possible “psychiatric cases” or not. Different authors have described the existence of three dimensions including successful coping, self-esteem and stress [17,18,19]. The GHQ-12 has been used in the Spanish National Health Survey (ENSE) to analyse the evolution of the population’s mental health, establish comparisons between groups or check the impact of specific situations, such as economic crises or pandemics [20].

Sedentary lifestyles and physical inactivity contribute to depression, anxiety, stress, and other symptomatology and mental disorders [21]. Research suggests that physical activity (PA) can lead to physiological changes that improve motivation, self-esteem, and lower stress levels. Exercise can reduce anxiety [22] and depression [23,24], even in extreme situations, such as during the COVID-19 pandemic [25]; thus, improving physical fitness can be a strategy to address the impact of an unhealthy lifestyle on mental health [26]. PA increases the endorphin and monoamine levels and decreases cortisol, improving patients’ mood [27]; exercise also increases the production of neurotransmitters [28,29] and attenuates the hypothalamic–pituitary–adrenal axis response to stress [29,30]. In addition, PA practice reduces blood pressure, improves cardiovascular fitness, and weight loss, and prevents chronic diseases such as cancer, diabetes, hypertension, obesity, osteoporosis, and cognitive impairment [31,32,33]. It also helps in mental and emotional aspects such as depression or anxiety [22], increased positive feelings associated with self-efficacy, and decreased negative thoughts [23,24]. Furthermore, PA practice often involves a social factor and the creation of routines that help people deal with depression, anxiety and stress [34].

Several studies have found associations between the PA practice and mental health improvements related to anxiety [22] and depression [34,35], reducing the risk of depression in the elderly [36]. Performing PA is also linked to increased positive feelings associated with self-efficacy and decreased negative thoughts [37,38]. Moreover, improvements in the muscular and skeletal system, cardiorespiratory, metabolic system [39] and decreased pain have been reported [40], all of which are key factors for physical and mental well-being and health. As far as we know, there are no studies published on the association between Physical Activity Level (PAL) and mental health in the Spanish population. The latest Spanish National Health Survey (ENSE 2017) [41], conducted by the Ministry of Health, Consumer Affairs and Social Welfare and the National Statistics Institute every five years, collected information on the health status of the Spanish population and was the last one before the pandemic. Therefore, this study aimed to analyse the associations between the different PAL and the mental health dimensions before the COVID-19 pandemic in Spain, providing a framework for future baselines to compare the effects of the pandemic on the associations between the variables described, based on new post-pandemic studies.

## 2. Materials and Methods

### 2.1. Study Design and Ethical Aspects

This manuscript reports a descriptive correlational study based on data from the Adult Questionnaire used in the ENSE 2017 to collect information on people from 15 to 103 years old [42], and whose interviews were held between October 2016 and 2017 by experienced surveyors.

Regulation 2016/679 of the European Parliament and of the Council of 27 April 2016 on the protection of individuals concerning the processing of personal data and on the free movement of personal data and derogating from Directive 95/46/EC [43] states that files for public use are not confidential; therefore, neither the application of data protection principles to anonymised information nor the approval of accredited ethics committees is required.

### 2.2. Participants

**Figure 1:**
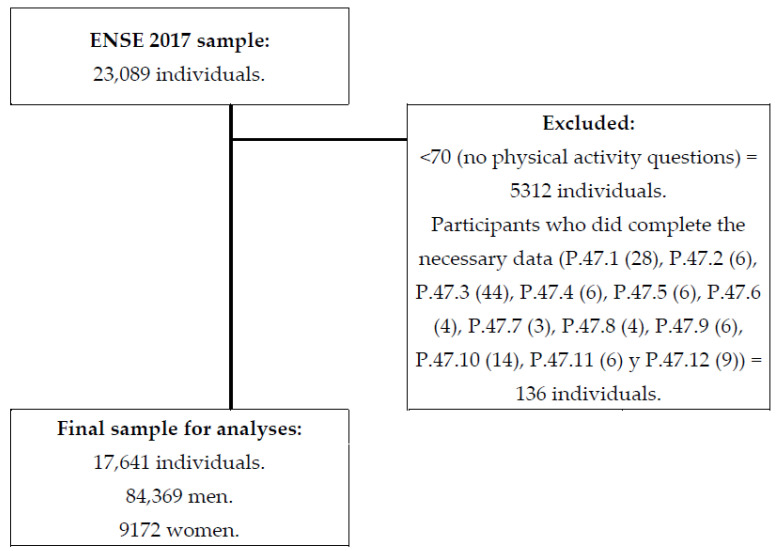
Chart outlining the study sample’s eligibility criteria.

A stratified three-phase random sampling was carried out in the Spanish population, considering people aged between 15 and 103 years, resulting in a 23,089 sample. A total of 10,595 men and 12,494 women were interviewed. In this research, 5312 individuals were excluded as the ENSE 2017 [41] did not ask about PA in the 69+ age group, and 136 individuals were excluded because they did not present complete data on the variables of interest for this study. Finally, the sample for our study was composed of 17,641 participants, including 8469 men and 9172 women (Figure 1).

### 2.3. Measures and Variables

The considered and created variables for this research were: 

Age: taken from the AGEa variable of the ENSE 2017 (years). 

Sex: taken from the SEXOa variable from the ENSE 2017 (male or female).

Mental health: the Spanish version of the Goldberg General Health Questionnaire (GHQ-12) was used. This questionnaire evaluates psychological health based on the answers to 12 items graded from 0 to 3, forming an overall index with the sum of all the answers. The total score ranges from 0 (the best condition) to 36 (the worst). The GHQ-12 presents high internal consistency (α = 0.86) [16]. The GHQ-12 is a self-administered screening test for non-psychotic psychiatric disorders, widely used in clinical settings and the general population both for its brevity and its psychometric characteristics. Although its factor structure has been a matter of debate, discussing whether it is composed of one factor, two (depression/anxiety and social dysfunction) or three factors, in this study we agree with the three-factor option [44] based on the factor analysis results: successful coping (FI), self-esteem (FII) and stress (FIII) [17,45]:

- Successful coping (FI): obtained by summing 6 items (1, 3, 4, 7, 8 and 12); scores ranged from 0 to 18 (0, the best; 18, the worst) and external validity of 0.82 with a *p*-value of 0.001.
- Self-esteem (FII): obtained by summing 4 items (6, 9, 10 and 11), with scores between 0 and 12 (0, the best; 12, the worst) and external validity of 0.70 with a *p*-value of 0.001.
- Stress (FIII): obtained by summing 3 items (2, 5 and 9), with scores between 0 and 9 (0, the best, 9, the worst) and external validity of 0.75, with a *p*-value of 0.001.

The Physical Activity Index (PAI) [46] was created by combining several PA factors with the answers obtained in the ENSE 2017. The factors were:

- Intensity: intense activity (10), moderate activity (5) and mild activity (0).
- Frequency: on the question “how many days did you practise intense and moderate PA?” the following values to the possible answers: “0” for zero days, “1” for one day per week, “2” for two or three days per week and “3” for more than three days per week.
- Duration: on the questions “how much time did you spend in total on intense PA? and, how much time did you spend in total on moderate PA?” a value of “1” was given for less than 30 min and “1.5” for 30 min or more.

So, the formula to find the PAI was = (intensity factor for intense activity * frequency factor for intense activity × duration factor for intense activity) + (intensity factor for moderate activity × frequency factor for moderate activity × duration factor for moderate activity). The factors were applied to intensity, frequency, and duration questions. PAI values range from 0 to 67.5 (a maximum of 45 for intense and 22.5 for moderate activities). Mild activities did not add value to the PAI. Thus, six PAL were established:

- “Inactive”: participants with PAI = 0 who answered the question “now think about how much time you spent walking in the last 7 days”, with “no day more than 10 min at a time”.
- “Insufficient”: participants with PAI = 0 who answered the question “now think about how much time you spent walking in the last 7 days” or stated, “at least one day more than 10 min consecutively”.
- “Low”: participants with a Physical Activity Index (PAI) score between 1 and 15 (75th percentile).
- “Medium”: individuals with a PAI score between 16 and 30 (90th percentile).
- “High”: participants with a PAI score between 31 and 45 (95th percentile).
- “Very high”: individuals with a PAI over 45 (values above the 95th percentile).

### 2.4. Statistical Analysis 

Statistical analysis was conducted using the Statistical Package for the Social Sciences (SPSS, Version 25, IBM SPSS, Armonk, NY, USA) software. Data distribution was analysed using the Kolmogorov–Smirnov test, and deciding to use non-parametric tests based on the results. Then, a descriptive statistical analysis was carried out to characterise the sample, by presenting age, mental health, successful coping, self-esteem, stress, and PAI variables using medians and interquartile ranges, complemented by means and standard deviations. The PAL was characterized using the absolute and relative frequencies of the population in its different levels, in total population and by sex. The Mann–Whitney U and the Chi-square tests for continuous and ordinal variables, respectively, were used to check potential differences between sexes and groups. The Kruskal–Wallis test was carried out to find differences at baseline between PAL and continuous variables from the GHQ-12, in addition to the post hoc Mann–Whitney U test to identify differences between the various PAL groups. Additionally, the effect size was calculated by using the z value (r = Z/√N), interpreted as 0.1 = small effect, 0.3 = medium and 0.5 = large effect [47]. Finally, a Spearman correlation study with the Bonferroni adjustment was carried out to analyse the associations between PAL and mental health dimensions. For all analyses, two-sided *p*-values ≤ 0.05 were considered statistically significant.

## 3. Results

Table 1 shows sociodemographic sample information (n = 17,641). Significant differences were found between general mental health status, self-esteem, successful coping, stress, PAI and PAL and sex of participants. Specifically, male participants presented higher scores in mental health and lower scores in PAI.

Significant correlations were found between PAL and mental health in the total population (Table 2). Significant differences were found between mental health and all its dimensions (GQH-12) in the “Inactive” and “Insufficient” PAL groups and between these two and the rest of the groups (*p* < 0.001), finding better mental health at higher PAL. Again, in the total population, a 3 points difference was found between the “Inactive” and “Medium”, “High”, and “Very high” groups’ median scores, representing a reduction of 27.3%. A 3.53 point difference was also found in the GHQ-12 mean scores between the “Inactive” and “Very high” groups, which means a reduction of 31.4%. Thus, a higher PAL was associated with better mental health, according to the GHQ-12 results.

In the men’s subgroup, differences were found between the “Inactive” and “Inadequate” PAL groups and between these and the other levels (Table 3). The median decreased by 11 points in the “Inactive” group and to 8 points in the “Medium”, “High”, and “Very high” groups, as in the total population. The groups’ mean scores on the GHQ-12 decreased as the level of PA increased, from a value of 12.04 in the “Inactive” group to 8.51 in the “Very high” group, with a difference of 3.53 points, which means a reduction of 29.3%.

In the women’s subgroup, significant differences were also found between the “Inactive” and “Low” PAL groups with the rest of the levels (Table 4). The median of the different groups decreased as the level of PA increased. The median decreased from 10 to 9 in the “Inactive” and “Low” groups, reaching 8 in the rest of the groups, representing a 20% decrease. Between the “Inactive” and “Poor” groups, the mean difference was 1.77 points on the GHQ-12, with the difference between the “Inactive” and “Very High” groups being 3.43 points less, representing a 27.6% reduction in score.

The Successful Coping factor (mental health factor 1) scored better with higher levels of PA in the total population. Significant differences were found between the “Inactive” and “Poor” groups and between these and the other groups (Table 5). The medians were the same for all groups. However, the groups’ median decreased as the PAL increased from 6.80 in the “Inactive” group to 5.76 in the “Very high” group.

The self-esteem factor (mental health factor 2) scored better at a higher PAL, with significant differences found between the “Inactive” and “Insufficient” groups and between these and the rest of the groups (Table 6). The median was 2 in the “Inactive” and “Insufficient” groups and 1 in the other groups. The group mean decreased as the PAL increased, with a reduction of 1.69 points between the “Inactive” and the “Very high” groups.

The Stress factor (in mental health factor 3) scored less as the PAL increased. No significant differences were found between the “Medium” and “High” levels, but significant differences were found between the other groups (Table 7).

As shown in Table 8, weak correlations between mental health and PAL were found in the total population as well as in the men and women subgroups. These correlations were inverse, with the GHQ-12 score decreasing as the PAL increased.

## 4. Discussion

### 4.1. Main Findings and Theoretical Applications

The main findings of this research are the associations between mental health and PAL in the Spanish population during the last pre-pandemic period analysed by the ENSE17 [41,42]. Thus, PAL seems to be linked with better mental health, coping, self-esteem, and stress levels. In addition, moderate and intense PAL showed stronger correlations with higher GHQ-12 scores. Although it is not the best option, in case of not being able to perform intense or moderate PA, walking seems to be a better alternative to physical inactivity for mental health care.

According to the data extracted from the ENSE 2017 and subsequent analysis, the Spanish population’s mental health appeared to be at a relatively good level. A median score of 9 was found, with significant differences between men (9) and women (10). Values in the Spanish population were below 12, a threshold that may imply emotional disorders [48,49]. However, significant differences were found in the GHQ-12 median values according to the PAL. Inactive people showed 11 points median on the GHQ-12. The median for people who at least walked was 10, 9 for people with a “Low” PAL, and 8 for the other levels, with medians decreasing by as much as 3.53 points when comparing inactive people and those with a “Very high” PAL. In this case, significant differences were found between the GHQ-12 medians of the groups with a different PAL. Our results recommend at least a “Medium” PAL to protect mental health. Similar findings were found in other studies, in which PA was associated with less psychological distress and improved mental health [50], and sedentary behaviours were associated with poorer mental health [51]. In the same sense, people who performed low PA, such as walking, also improved successful coping, stress, or self-esteem, although lower than those who performed moderate and intense PA. The groups with higher PAL presented better values in the three mental health dimensions and significant differences between sedentary people and those who only walked. Therefore, moderate and intense PA is recommended for mental health care according to the GHQ-12 results. Other studies have indicated that higher PAL protects people from depression [36], anxiety and other disorders [22] compared with those with lower PAL.

These associations were also analysed during the COVID-19 pandemic, showing that people who exercised daily had fewer somatisation symptoms, lower stress, and better sleep levels than those who did not [52,53]. In addition, appropriate PA helped people to release psychological tension during confinement [54]. In this regard, symptoms related to anxiety, depression and stress were found in people with a lack of PA and a sedentary lifestyle [55,56,57,58]. Furthermore, the pandemic has negatively affected PAL, particularly in outdoor activities, which have been shown to have protective effects on well-being [53].

In the analysis of the dimensions, Self-esteem was the dimension that benefited the most from higher PAL, both in the total population and in the subgroups divided by sex. The “Inactive” PAL group had a 3.01 mean, compared to the 1.32 in the “Very high” group, a 56.1% reduction in the FII score. Increased self-confidence could be one of the main ways PA helped with mental health care in the Spanish population. However, the Stress dimension mean decreased from 3.13 in the “Inactive” to 1.82, representing a 41.8% decrease. The Successful Coping dimension was the least benefited by higher PAL: the mean decreased from 6.80 in the “Inactive” to 5.76 in the “Very high” group, a 15.3% decrease. Thus, better mental health due to increased PA could be due to increased self-esteem and reduced stress, with minor improvement in successful coping. In this line, several studies suggest that PA improves self-efficacy and coping with new challenges that build confidence and self-esteem [59,60]. Walking ≥105 min/week compared to <105 min/week was significantly and inversely associated with stress and anxiety [61]. In addition, self-reported PA was associated with lower subjective stress levels; even low/moderate daily PA was associated with significantly lower stress levels [62]. In this sense, PA could have a stress-reducing effect [63].

During the COVID-19 pandemic, stress levels and sleep quality improved in people who exercised regularly, showing decreased risk of depressive and anxiety symptoms in participants who reported ≥30 min of moderate–vigorous PA/day [52]. In contrast, participants who spent ≥10 h per day in sedentary activities were more likely to develop depressive symptoms [23]. Therefore, PA and emotional well-being associations keep before and during the COVID-19 pandemic. Concerning the sex subgroups, inactive women obtained a GHQ-12 median score of 12, the threshold for emotional distress, while this value decreased to 8 in women who performed higher PA. Also, in women, the PAL and self-esteem were related: higher self-esteem was reported at moderate and high PAL [64]. Inactive men had a self-esteem score of 10, which decreased to 8 in those who performed greater PA. According to our data, PA duration and intensity had a more significant influence on mental health in women than in men. Although there were differences between inactive women and those who performed light-intensity PA, those who performed <1 h, 1–2 h, or ≥3 h/week were more likely to develop anxiety. No inverse associations were observed between men and women [65].

### 4.2. Practical Applications and Future Lines

This study provides a baseline to analyse potential changes in the PA–mental health associations in the Spanish population once the future ENSE is published. As these surveys are conducted every 5 years, the next is expected to be published in 2023, with data from 2022, favouring future research on the impact of the pandemic on these associations.

Although the study design does not allow for cause–effect relations, longitudinal studies will provide the necessary information for the development of PA interventions and guidelines as a tool for mental health disease prevention and treatment. 

### 4.3. Strengths and Limitations

The use of the ENSE 2017 is an excellent example of a nationally representative survey, and the sample size is the study’s main strength.

However, some limitations must be mentioned: (1) as this is a cross-sectional study, it is not possible to establish cause–effect associations; (2) the GHQ-12 questionnaire is a screening instrument that may lead to an overestimation of mental health problems; moreover, some of the limitations of self-report questionnaires include social desirability and response bias, or item clarity, which may affect the validity and reliability of the tool [66]; (3) a 24 h compositional analysis, including objective PA parameters, was not included, so the PAI was determined using the parameters indicated in the ENSE 2017; (4) the survey did not collect PA data from people over 69 years of age, which could have affected the analysis: (5) changes in the future ENSE methodology could prevent comparisons with data obtained in this study.

## 5. Conclusions

This research found that PAL was positively related to mental health in the Spanish population before the COVID-19 pandemic. In addition, moderate and intense PAL showed stronger correlations with higher GHQ-12 scores.

These results need to be confirmed with longitudinal studies to recommend PA programs as a valid alternative to promote mental health.

## Figures and Tables

**Table 1:** Goldberg General Health Questionnaire and physical activity level according to age, mental health, and its dimensions.

Age (Years)	Total (N = 17,641)	Men	Women (N = 9172)	*p*-Value
Participants				
Median (IQR)	47 (21)	47 (21)	47 (21)	0.274 a
Mean (SD)	45.8 (14.1)	45.7 (14.1)	46.0 (14.1)	
GHQ-12 total scores (Mental health)				
Median (IQR)	9 (5)	9 (6)	10 (5)	<0.001 a
Mean (SD)	10.1 (4.7)	9.7 (4.5)	10.5 (4.9)	
FI: Successful coping				
Median (IQR)	6 (0)	6 (0)	6 (0)	<0.001 a
Mean (SD)	6.2 (1.8)	6.2 (1.7)	6.3 (1.8)	
FII: Self-esteem				
Median (IQR)	1 (4)	1 (3)	2 (4)	<0.001 a
Mean (SD)	2.1 (1.8)	1.9 (2.2)	2.2 (2.4)	
FIII: Stress				
Median (IQR)	3 (2)	2 (3)	3 (3)	<0.001 a
Mean (SD)	2.5 (2.1)	2.2 (2)	2.7 (2.1)	
Physical Activity Index (PAI)				
Median (IQR)	0 (22.5)	0 (30)	0 (15)	<0.001 a
Mean (SD)	11.8 (17.6)	14.6 (19.4)	9.2 (15.3)	
Physical Activity Level (%)				
Inactive (PAI = 0)	2497 (14.2%)	1157 (13.7%)	1340 (14.7%)	<0.001 b
Insufficient (PAI = 0)	8005 (45.6%)	3364 (40%)	4641 (50.7%)	
Low (PAI = 1–15)	2417 (13.8%)	1116 (13.3%)	1301 (14.2%)	
Medium (PAI = 16–30)	2436 (13.9%)	1329 (15.8%)	1107 (12.1%)	
High (PAI = 31–45)	1452 (8.3%)	941 (11.2%)	511 (5.6%)	
Inactive (PAI = +45)	754 (4.3%)	508 (6%)	246 (2.7%)	

IQR: interquartile range; SD: standard deviation; GHQ-12: Goldberg’s General Health Questionnaire, scores between 0 and 36; FI Successful Coping: scores between 0 and 18. 0, the best coping and 18, the worst; FII Self-esteem: scores 0–9, 0 the best self-esteem and 9, the worst); FIII Stress: scores between 0 and 9. 0, no stress and 9, very stressed; PAI: Physical Activity Index, considering only intense and moderate physical activity, scores 0–67.5; Inactive: PAI = 0, reporting not going out for more than 10 min at a time; Insufficient: PAI = 0, reporting to walk more than 10 min; a: *p*-value from Mann–Whitney U test; b: *p*-value from Chi-square test.

**Table 2:** Using the Goldberg General Health Questionnaire and the physical activity level comparison, mental health outcomes were measured.

PAI		GHQ-12	PAI	Medians Diff.	Means Diff.	*p* *	*p* **	Effect Size
Inactive	Median	11	Insufficient	1	1.64	<0.001	<0.001	0.119
	IQR	5	Low	2	2.63		<0.001	0.241
	Mean	12.04	Medium	3	2.91		<0.001	0.276
	SD	6.11	High	3	3.20		<0.001	0.293
			Very high	3	3.53		<0.001	0.294
Insufficient	Median	10	Inactive	−1	−1.64	<0.001	<0.001	0.119
	IQR	5	Low	1	0.99		<0.001	0.088
	Mean	10.40	Medium	2	1.27		<0.001	0.123
	SD	4.73	High	2	1.56		<0.001	0.125
			Very high	2	1.89		<0.001	0.125
Low	Median	9	Inactive	−2	−2.63	<0.001	<0.001	0.241
	IQR	5	Insufficient	−1	−0.99		<0.001	0.088
	Mean	9.41	Medium	1	0.28		0.002	0.045
	SD	3.96	High	1	0.57		<0.001	0.071
			Very high	1	0.90		<0.001	0.109
Medium	Median	8	Inactive	−3	−2.91	<0.001	<0.001	0.276
	IQR	5	Insufficient	−2	−1.27		<0.001	0.123
	Mean	9.13	Low	−1	−0.28		0.002	0.045
	SD	3.93	High	0	0.29		0.090	0.027
			Very high	0	0.62		<0.001	0.069
High	Median	8	Inactive	−3	−3.20	<0.001	<0.001	0.293
	IQR	5	Insufficient	−2	−1.56		<0.001	0.125
	Mean	8.84	Low	−1	−0.57		<0.001	0.071
	SD	3.57	Medium	0	−0.29		0.090	0.027
			Very high	0	0.33		0.015	0.051
Very high	Median	8	Inactive	−3	−3.53	<0.001	<0.001	0.294
	IQR	4	Insufficient	−2	−1.89		<0.001	0.125
	Mean	8.51	Low	−1	−0.90		<0.001	0.109
	SD	3.62	Medium	0	−0.62		<0.001	0.069
			High	0	−0.33		0.015	0.051

IQR: interquartile range; SD: standard deviation; GHQ-12: Goldberg’s General Health Questionnaire, scores between 0 and 36; Medians Diff: mental health medians differences for every physical activity level; Means diff: between mental health means differences for every physical activity level; *p* * Kruskal–Wallis value: mental health measured by GHQ-12 as response and physical activity level as a factor; PAI: Physical Activity Index, considering only intense and moderate physical activity, scores 0–67.5; Inactive: PAI = 0, reporting not going out for more than 10 min at a time; Insufficient: PAI = 0, reporting to walk more than 10 min; Low: PAI between 1 and 15; Medium: PAI between 16 and 30; High: PAI between 31 and 45; Very high: PAI > 45; ** *p* Mann–Whitney U test: resulting from the mental health median comparison for every physical activity level.

**Table 3:** Mental health outcomes using the Goldberg General Health Questionnaire and the physical activity level comparison in men.

PAI		GHQ-12	PAI	Medians Diff.	Means Diff.	p *	p **	Effect Size
Inactive	Median	10	Insufficient	1	1.56	<0.001	<0.001	0.112
	IQR	5	Low	2	2.51		<0.001	0.226
	Mean	11.57	Medium	2	2.56		<0.001	0.241
	SD	6.01	High	2	2.92		<0.001	0.273
			Very high	2	3.31		<0.001	0.302
Insufficient	Median	9	Inactive	−1	−1.56	<0.001	<0.001	0.112
	IQR	5	Low	1	0.95		<0.001	0.086
	Mean	10.01	Medium	1	1		<0.001	0.108
	SD	4.7	High	1	1.36		<0.001	0.126
			Very high	1	1.76		<0.001	0.144
Low	Median	8	Inactive	−2	−2.51	<0.001	<0.001	0.226
	IQR	5	Insufficient	−1	−0.95		<0.001	0.086
	Mean	9.06	Medium	0	0.05		0.255	0.024
	SD	3.68	High	0	0.41		0.015	0.055
			Very high	0	0.81		<0.001	0.113
Medium	Median	8	Inactive	−2	−2.56	<0.001	<0.001	0.241
	IQR	5	Insufficient	−1	−1		<0.001	0.108
	Mean	9.01	Low	0	−0.05		0.255	0.024
	SD	3.96	High	0	0.36		0.164	0.029
			Very high	0	0.76		<0.001	0.085
High	Median	8	Inactive	−2	−2.92	<0.001	<0.001	0.273
	IQR	5	Insufficient	−1	−1.36		<0.001	0.126
	Mean	8.65	Low	0	−0.41		0.015	0.055
	SD	3.38	Medium	0	−0.36		0.090	0.029
			Very high	0	0.40		0.014	0.064
Very high	Median	8	Inactive	−2	−3.31	<0.001	<0.001	0.302
	IQR	4	Insufficient	−1	−1.76		<0.001	0.144
	Mean	8.25	Low	0	−0.81		<0.001	0.081
	SD	3.35	Medium	0	−0.76		<0.001	0.085
			High	0	−0.40		0.014	0.064

IQR: interquartile range; SD: standard deviation; GHQ-12: Goldberg’s General Health Questionnaire, scores between 0 and 36; Medians Diff: mental health medians differences for every physical activity level; Means diff: between mental health means differences for every physical activity level; *p* * Kruskal–Wallis value: mental health measured by GHQ-12 as response and physical activity level as a factor; PAI: Physical Activity Index, considering only intense and moderate physical activity, scores 0–67.5; Inactive: PAI = 0, reporting not going out for more than 10 min at a time; Insufficient: PAI = 0, reporting to walk more than 10 min; Low: PAI between 1 and 15; Medium: PAI between 16 and 30; High: PAI between 31 and 45; Very high: PAI > 45; ** *p* Mann–Whitney U test: resulting from the mental health median comparison for every physical activity level.

**Table 4:** Mental health outcomes using the Goldberg General Health Questionnaire and the physical activity level comparison in women.

PAI		GHQ-12	PAI	Medians Diff.	Means Diff.	p *	p **	Effect Size
Inactive	Median	12	Insufficient	1	1.77	<0.001	<0.001	0.130
	IQR	6	Low	2	2.74		<0.001	0.255
	Mean	12.45	Medium	2	3.17		<0.001	0.300
	SD	6.07	High	2	3.26		<0.001	0.275
			Very high	2	3.43		<0.001	0.240
Insufficient	Median	10	Inactive	−1	−1.77	<0.001	<0.001	0.130
	IQR	5	Low	1	0.97		<0.001	0.085
	Mean	10.68	Medium	1	1.40		<0.001	0.122
	SD	4.88	High	1	1.49		<0.001	0.098
			Very high	1	1.66		<0.001	0.083
Low	Median	9	Inactive	−2	−2.74	<0.001	<0.001	0.255
	IQR	5	Insufficient	−1	−0.97		<0.001	0.085
	Mean	9.71	Medium	0	0.43		0.006	0.056
	SD	4.16	High	0	0.52		<0.001	0.059
			Very high	0	0.71		<0.001	0.068
Medium	Median	9	Inactive	−2	−3.17	<0.001	<0.001	0.300
	IQR	5	Insufficient	−1	−1.40		<0.001	0.122
	Mean	9.28	Low	0	−0.43		0.006	0.056
	SD	3.89	High	0	0.09		0.670	0.010
			Very high	0	0.26		0.238	0.030
High	Median	8	Inactive	−2	−3.26	<0.001	<0.001	0.275
	IQR	5	Insufficient	−1	−1.49		<0.001	0.098
	Mean	9.19	Low	0	−0.52		<0.001	0.059
	SD	3.89	Medium	0	−0.09		0.670	0.010
			Very high	0	0.17		0.458	0.024
Very high	Median	8	Inactive	−2	−3.43	<0.001	<0.001	0.240
	IQR	4	Insufficient	−1	−1.66		<0.001	0.083
	Mean	9.02	Low	0	−0.71		<0.001	0.047
	SD	4.06	Medium	0	−0.26		0.238	0.030
			High	0	−0.17		0.458	0.024

IQR: interquartile range; SD: standard deviation; GHQ-12: Goldberg’s General Health Questionnaire, scores between 0 and 36; Medians Diff: mental health medians differences for every physical activity level; Means diff: between mental health means differences for every physical activity level; *p* * Kruskal–Wallis value: mental health measured by GHQ-12 as response and physical activity level as a factor; PAI: Physical Activity Index, considering only intense and moderate physical activity, scores 0–67.5; Inactive: PAI = 0, reporting not going out for more than 10 min at a time; Insufficient: PAI = 0, reporting to walk more than 10 min; Low: PAI between 1 and 15; Medium: PAI between 16 and 30; High: PAI between 31 and 45; Very high: PAI > 45; ** *p* Mann–Whitney U test: resulting from the mental health median comparison for every physical activity level.

**Table 5:** Mental health factor I, “Successful coping”, and the physical activity level comparison.

PAI		FI	PAI	Medians Diff.	Means Diff.	p *	p **	Effect Size
Inactive	Median	6	Insufficient	0	0.50	<0.001	<0.001	0.066
	IQR	1	Low	0	0.81		<0.001	0.168
	Mean	6.80	Medium	0	0.85		<0.001	0.175
	SD	2.46	High	0	0.94		<0.001	0.197
			Very high	0	1.04		<0.001	0.181
Insufficient	Median	6	Inactive	0	−0.50	<0.001	<0.001	0.066
	IQR	0	Low	0	0.31		<0.001	0.084
	Mean	6.30	Medium	0	0.35		<0.001	0.091
	SD	1.71	High	0	0.44		<0.001	0.103
			Very high	0	0.54		<0.001	0.087
Low	Median	6	Inactive	0	−0.81	<0.001	<0.001	0.168
	IQR	0	Insufficient	0	−0.31		<0.001	0.084
	Mean	5.99	Medium	0	0.04		0.569	0.008
	SD	1.40	High	0	0.13		0.007	0.043
			Very high	0	0.23		0.005	0.050
Medium	Median	6	Inactive	0	−0.85	<0.001	<0.001	0.175
	IQR	0	Insufficient	0	−0.35		<0.001	0.091
	Mean	5.95	Low	0	−0.04		0.569	0.008
	SD	1.41	High	0	0.09		0.029	0.035
			Very high	0	0.19		0.015	0.043
High	Median	6	Inactive	0	−0.94	<0.001	<0.001	0.197
	IQR	0	Insufficient	0	−0.44		<0.001	0.103
	Mean	5.86	Low	0	−0.13		0.007	0.043
	SD	1.34	Medium	0	−0.09		0.029	0.035
			Very high	0	0.10		0.517	0.014
Very high	Median	6	Inactive	0	−1.04	<0.001	<0.001	0.181
	IQR	0	Insufficient	0	−0.54		<0.001	0.087
	Mean	5.76	Low	0	−0.23		0.005	0.050
	SD	1.42	Medium	0	−0.19		0.015	0.043
			High	0	−0.10		0.517	0.014

IQR: interquartile range; SD: standard deviation; FI, from the Goldberg’s General Health Questionnaire, scores between 0 and 18, 0 being the best coping, and 18, the worst; Medians Diff: mental health medians differences for every physical activity level; Means diff: between mental health means differences for every physical activity level; *p* * Kruskal–Wallis value: mental health measured by GHQ-12 as response and physical activity level as a factor; PAI: Physical Activity Index, considering only intense and moderate physical activity, scores 0–67.5; Inactive: PAI = 0, reporting not going out for more than 10 min at a time; Insufficient: PAI = 0, reporting to walk more than 10 min; Low: PAI between 1 and 15; Medium: PAI between 16 and 30; High: PAI between 31 and 45; Very high: PAI > 45; ** *p* Mann–Whitney U test: resulting from the mental health median comparison for every physical activity level.

**Table 6:** Mental health factor II, “Self-esteem” and the physical activity level comparison.

PAI		FII	PAI	Medians Diff.	Means Diff.	*p* *	*p* **	Effect Size
Inactive	Median	2	Insufficient	0	0.83	<0.001	<0.001	0.129
	IQR	3	Low	1	1.28		<0.001	0.245
	Mean	3.01	Medium	1	1.40		<0.001	0.273
	SD	2.83	High	1	1.58		<0.001	0.296
			Very high	1	1.69		<0.001	0.282
Insufficient	Median	2	Inactive	0	−0.83	<0.001	<0.001	0.129
	IQR	4	Low	1	0.45		<0.001	0.081
	Mean	2.18	Medium	1	0.57		<0.001	0.108
	SD	2.32	High	1	0.75		<0.001	0.116
			Very high	1	0.86		<0.001	0.108
Low	Median	1	Inactive	−1	−1.28	<0.001	<0.001	0.245
	IQR	3	Insufficient	−1	−0.45		<0.001	0.081
	Mean	1.73	Medium	0	0.12		0.018	0.034
	SD	2.00	High	0	0.30		<0.001	0.065
			Very high	0	0.41		<0.001	0.086
Medium	Median	1	Inactive	−1	−1.40	<0.001	<0.001	0.273
	IQR	3	Insufficient	−1	−0.57		<0.001	0.108
	Mean	1.61	Low	0	−0.12		0.018	0.034
	SD	2.00	High	0	0.18		0.051	0.031
			Very high	0	0.29		0.002	0.055
High	Median	1	Inactive	−1	−1.58	<0.001	<0.001	0.296
	IQR	2	Insufficient	−1	−0.75		<0.001	0.116
	Mean	1.43	Low	0	−0.30		<0.001	0.065
	SD	1.78	Medium	0	−0.18		0.051	0.031
			Very high	0	0.11		0.125	0.033
Very high	Median	1	Inactive	−1	−1.69	<0.001	<0.001	0.282
	IQR	2	Insufficient	−1	−0.86		<0.001	0.108
	Mean	1.32	Low	0	−0.41		<0.001	0.086
	SD	1.78	Medium	0	−0.29		0.002	0.055
			High	0	−0.11		0.125	0.033

IQR: interquartile range; SD: standard deviation; FII, Self-esteem, from the Goldberg’s General Health Questionnaire, scores between 0 and 9, 0 being for the best self-esteem and 9, the worst; Medians Diff: mental health medians differences for every physical activity level; Means diff: between mental health means differences for every physical activity level; *p* * Kruskal–Wallis value: mental health measured by GHQ-12 as response and physical activity level as a factor; PAI: Physical Activity Index, considering only intense and moderate physical activity, scores 0–67.5; Inactive: PAI = 0, reporting not going out for more than 10 min at a time; Insufficient: PAI = 0, reporting to walk more than 10 min; Low: PAI between 1 and 15; Medium: PAI between 16 and 30; High: PAI between 31 and 45; Very high: PAI > 45; ** *p* Mann–Whitney U test: resulting from the mental health median comparison for every physical activity level.

**Table 7:** Mental health factor III, “Stress” and the physical activity level comparison.

PAI		FIII	PAI	Medians Diff.	Means Diff.	*p* *	*p* **	Effect Size
Inactive	Median	3	Insufficient	0	0.51	<0.001	<0.001	0.099
	IQR	2	Low	1	0.87		<0.001	0.203
	Mean	3.13	Medium	1	1.04		<0.001	0.247
	SD	2.21	High	1	1.11		<0.001	0.254
			Very high	1	1.31		<0.001	0.269
Insufficient	Median	3	Inactive	0	−0.51	<0.001	<0.001	0.099
	IQR	3	Low	1	0.36		<0.001	0.071
	Mean	2.62	Medium	1	0.53		<0.001	0.111
	SD	2.08	High	1	0.60		<0.001	0.106
			Very high	1	0.80		<0.001	0.115
Low	Median	2	Inactive	−1	−0.87	<0.001	<0.001	0.203
	IQR	2	Insufficient	−1	−0.36		<0.001	0.071
	Mean	2.26	Medium	0	0.17		<0.001	0.051
	SD	1.91	High	0	0.24		<0.001	0.065
			Very high	0	0.44		<0.001	0.110
Medium	Median	2	Inactive	−1	−1.04	<0.01	<0.001	0.247
	IQR	3	Insufficient	−1	−0.53		<0.001	0.111
	Mean	2.09	Low	0	−0.17		<0.001	0.051
	SD	1.91	High	0	0.07		0.343	0.015
			Very high	0	0.27		0.001	0.066
High	Median	2	Inactive	−1	−1.11	<0.001	<0.001	0.254
	IQR	3	Insufficient	−1	−0.60		<0.001	0.106
	Mean	2.02	Low	0	−0.24		<0.001	0.065
	SD	1.85	Medium	0	−0.07		0.343	0.015
			Very high	0	0.21		0.006	0.058
Very high	Median	2	Inactive	−1	−0.93	<0.001	<0.001	0.269
	IQR	3	Insufficient	−1	−0.93		<0.001	0.115
	Mean	1.82	Low	0	−0.45		<0.001	0.110
	SD	1.83	Medium	0	−0.28		<0.001	0.066
			High	0	−0.03		0.006	0.058

IQR: interquartile range; SD: standard deviation; FIII, Stress, from the Goldberg’s General Health Questionnaire, scores between 0 and 9, 0 being for the best self-esteem and 9, the worst; Medians Diff: mental health medians differences for every physical activity level; Means diff: between mental health means differences for every physical activity level; *p* * Kruskal–Wallis value: mental health measured by GHQ-12 as response and physical activity level as a factor; PAI: Physical Activity Index, considering only intense and moderate physical activity, scores 0–67.5; Inactive: PAI = 0, reporting not going out for more than 10 min at a time; Insufficient: PAI = 0, reporting to walk more than 10 min; Low: PAI between 1 and 15; Medium: PAI between 16 and 30; High: PAI between 31 and 45; Very high: PAI > 45; ** *p* Mann–Whitney U test: resulting from the mental health median comparison for every physical activity level.

**Table 8:** Associations between Goldberg’s General Health Questionnaire and their subscales and the physical activity level.

Physical Activity Level						
Variables	Total		Men		Women	
	rho	*p*	rho	*p*	rho	*p*
Mental health (GHQ-12)	−0.200	<0.001	−0.187	<0.001	−0.194	<0.001
FI: Successful Coping	−0.141	<0.001	−0.139	<0.001	−0.136	<0.001
FII: Self-esteem	−0.192	<0.001	−0.179	<0.001	−0.189	<0.001
FIII: Stress	−0.175	<0.001	−0.159	<0.001	−0.166	<0.001

Rho: Spearman’s correlation coefficient with Bonferroni correction.

## Data Availability

Datasets will be available under reasonable request.
